# Safety Profile of Medications for Allergic Rhinitis: A Meta-Epidemiological Analysis of Completed RCTs from ClinicalTrials.gov

**DOI:** 10.3390/pharmaceutics18050581

**Published:** 2026-05-08

**Authors:** Ivan Paladin, Mirko Maglica, Marin Gudelj, Emilija Krezo Šljivić, Franko Batinović, Darko Batistić, Shelly Melissa Pranić

**Affiliations:** 1 Department of ENT and Head and Neck Surgery, University Hospital of Split, 21000 Split, Croatia; ivan.paladin@gmail.com (I.P.); marin.gudelj8@gmail.com (M.G.); emilijakrezo@gmail.com (E.K.Š.); fbatinovic1@gmail.com (F.B.); batisticdarko@gmail.com (D.B.); 2 Department of Public Health, University of Split School of Medicine, 21000 Split, Croatia

**Keywords:** allergic rhinitis, drug safety, adverse events, intranasal corticosteroids, sublingual immunotherapy

## Abstract

**Background**: Allergic rhinitis (AR) is commonly treated with intranasal and oral pharmacotherapy or allergen immunotherapy (AIT), each associated with distinct safety considerations. This study aimed to systematically evaluate and compare the safety profiles of these therapeutic approaches by analysing adverse events (AEs) reported in completed randomised controlled trials (RCTs). **Methods**: A meta-epidemiological analysis was conducted using completed RCTs registered in ClinicalTrials.gov up to 20 October 2023. Trials investigating intranasal drugs, oral medications, or AIT for AR were identified using predefined search terms. Adverse events were manually extracted and categorised according to treatment class and dosage. Other adverse events (OAEs) were classified using Common Terminology Criteria for Adverse Events version 5.0 (CTCAE v5.0) and Medical Dictionary for Regulatory Activities (MedDRA) terminology. Meta-analyses compared OAE incidence across treatment groups, including standard-dose, higher-dose, and placebo arms. **Results**: A total of 216 RCTs were included. Intranasal therapies accounted for 55.56% of trials, predominantly intranasal corticosteroids (INCS) and intranasal antihistamines (INAH). OAE incidence was 16.37% for INCS, 29.43% for INAH, and 8.71% for combination therapy. INAH was associated with higher rates of dysgeusia and nasal discomfort, while higher INCS doses were linked to an increased risk of urinary tract infections. AIT trials comprised 22.69% of studies and demonstrated higher OAE rates, particularly for sublingual immunotherapy (64.96%), followed by subcutaneous (53.98%) and intralymphatic immunotherapy (62.50%). Oropharyngeal AEs were most frequent with sublingual immunotherapy. Oral medications (18.06%) showed the lowest OAE incidence, with upper respiratory tract infections occurring more frequently with oral antihistamines. **Conclusions**: Among intranasal therapies, INCS demonstrated the most favourable safety profile. Sublingual immunotherapy was associated with a higher frequency of OAEs compared with other AIT modalities. Combination oral antihistamine and leukotriene receptor antagonist therapy appeared to be the safest oral treatment option. Further well-designed studies are needed to refine comparative safety assessments across AR treatments.

## 1. Introduction

Allergic rhinitis (AR) is one of the most common chronic respiratory conditions worldwide, affecting up to 10–30% of the global population [1,2]. It is associated with a substantial burden on quality of life, including sleep disturbance, impaired cognitive function, and reduced daily performance [1]. AR also negatively impacts work productivity and school performance, contributing to significant indirect costs [3]. In addition, AR is frequently associated with comorbid conditions such as asthma, sinusitis, and conjunctivitis, further increasing its clinical and economic burden [1].

The fundamental therapeutic strategies for managing AR include intranasal corticosteroids (INCS) and antihistamines (INAH), either alone or in combination, oral antihistamines (OAH) and leukotriene receptor antagonists (LTRA), also alone or in combination, as well as allergen immunotherapy (AIT). Each strategy has specific risks and benefits [4,5,6]. Although these medications are generally effective in controlling AR symptoms [7,8,9,10,11,12], their safety and potential adverse events (AEs) are key factors in making therapeutic decisions [13].

The safety profiles of pharmacological treatments for AR differ across drug classes and represent an important factor in treatment selection. INCS are generally well tolerated but may be associated with local AEs, while INAH are often linked to taste disturbances and local irritation. AIT is associated with both local and, less frequently, systemic reactions, whereas oral therapies are typically well tolerated but may carry class-specific risks [1,14].

Drug safety for AR is assessed using data from randomised controlled trials (RCTs), which represent the gold standard for evaluating drug efficacy and safety [15]. RCTs allow control over factors that may influence outcomes and provide data on the frequency and severity of AEs compared to placebo or other therapies [16].

This study aims to analyse the safety profiles of key pharmacological treatments for AR by examining data on reported other adverse events (OAEs) from completed RCTs registered on ClinicalTrials.gov, with the goal of informing clinical practice and optimising therapeutic decision-making. Despite the availability of numerous RCTs and individual safety reports of safety outcomes, there is a lack of comprehensive and standardised analyses focusing on AE reporting across different pharmacological classes.

It was designed as a meta-epidemiological analysis, aiming to evaluate patterns in AE reporting across RCTs rather than to estimate pooled treatment effects. This approach enables systematic comparison of safety profiles across interventions and provides insight into the consistency and reporting of AEs across trials using a unified data source.

## 2. Materials and Methods

### 2.1. Search Strategy and Reporting

The study was conducted as a meta-epidemiological analysis evaluating data from completed RCTs on AR, registered in the ClinicalTrials.gov registry from inception and updated no later than 20 October 2023. We followed reporting guidelines for meta-epidemiological studies, adapted from PRISMA (Preferred Reporting Items for Systematic Reviews and Meta-Analyses) guidelines [17]. The completed PRISMA checklist is provided as a Supplementary File. On 23 October 2023, IP searched for completed RCTs in ClinicalTrials.gov using the keywords “allergic rhinitis,” “nasal allergies,” “rhinoconjunctivitis,” “hay fever,” and “atopic rhinitis.” The exact search query used in ClinicalTrials.gov was: (“allergic rhinitis” OR “nasal allergies” OR “rhinoconjunctivitis” OR “hay fever” OR “atopic rhinitis”). No MeSH terms or additional intervention-based filters were applied, and the search was conducted using condition-based keywords only. The same author downloaded the retrieved records as a .csv file, which included basic trial characteristics. This study was registered in the Open Science Framework (OSF) repository, and all data underlying this study are publicly accessible via the OSF repository (https://doi.org/10.17605/OSF.IO/4J3GF, accessed on 2 June 2025). Consistent with this approach, the study focuses on identifying aggregate patterns in AE reporting across trials rather than estimating intervention-specific effect sizes.

### 2.2. Eligibility Criteria

Eligible studies were completed RCTs registered on ClinicalTrials.gov that assessed either the efficacy or the safety of pharmacological interventions for AR. Inclusion was determined through manual review of each study’s outcome measures, independently conducted by IP and MM. Trials were included only if the predefined search terms appeared in the study title or in the “Conditions” field of the registry entry. We excluded trials that were non-randomised, ongoing, unrelated to AR, investigated a device, or were terminated.

### 2.3. Data Extraction

The extracted data from ClinicalTrials.gov were entered into a Microsoft Excel table. AEs presented in tabular form within each ClinicalTrials.gov registry entry were manually extracted and categorised according to the studied drug and treatment group (i.e., intervention, active control, or placebo). Additional trial-level data, including treatment duration, dosing frequency, and participants’ mean age, sex, and race, were manually extracted from each registry entry. Data extraction was performed using a predefined extraction sheet developed in Microsoft Excel. The extraction was performed by four authors (MG, EKŠ, FB, and DB), and subsequently reviewed and verified by IP for accuracy and consistency. Prior to full data extraction, all extractors participated in a training session using a sample of ten randomly selected RCTs to ensure consistency and a shared understanding of AE categorisation. This calibration process included joint discussions and minor modifications to the data extraction sheet to improve clarity and usability. After harmonising the extraction procedure, the remaining studies were evenly distributed among the four extractors. Any uncertainties or disagreements were resolved through group discussion and consensus. IN medications was divided into INCS, INAH, and their combination; AIT was divided into sublingual immunotherapy (SLIT), subcutaneous immunotherapy (SCIT), and intralymphatic immunotherapy (ILIT); and oral medications were divided into OAH, LTRA, and their combination. Subgroup classification was based on pharmacological mechanisms and clinical use patterns to enable meaningful comparison of safety profiles across therapeutically relevant categories. Each subgroup was further classified based on whether participants received the investigated drug, a higher dose of the same drug as an active control, or a placebo. This information was extracted manually from the ‘Arms and Interventions’ and ‘Detailed Description’ sections of each ClinicalTrials.gov record. The categorisation was performed by the same four authors (MG, EKŠ, FB, and DB) and verified by IP. In cases where dose classification was unclear, group consensus was reached following discussion. Subsequent analyses were presented according to this classification. To ensure consistency across studies with heterogeneous reporting formats, all AEs were standardised using a predefined classification framework to enable consistent comparison across trials.

An AE was defined as any unfavourable medical occurrence in a participant, including any abnormal sign, symptom, or disease, temporally associated with participation in the study, irrespective of whether it was considered related to the research. Serious adverse events (SAEs) were defined as AEs that result in death, are life-threatening, require or prolong hospitalisation, cause persistent/significant disability, or involve a congenital anomaly or birth defect. OAEs refer to all reported AEs that do not meet the criteria for seriousness but are nonetheless documented in the ClinicalTrials.gov registry, including both common and uncommon side effects [18]. In our study, OAEs were the primary focus, as they were more consistently reported across studies, whereas SAEs were often infrequent or absent. As ClinicalTrials.gov does not indicate whether AEs are treatment-related (TRAE) or treatment-emergent (TEAE), all reported AEs were extracted to minimise reporting bias. Meta-analyses were performed comparing the investigated treatment, active control, and placebo groups, enabling consistent and comparable assessment of AE frequency across studies.

OAEs were tabulated according to the Common Terminology Criteria for Adverse Events version 5.0 (CTCAE v5.0) [19], including their MedDRA (Medical Dictionary for Regulatory Activities) “Preferred term” and distribution according to MedDRA System Organ Classes (SOCs) [20]. Additionally, OAEs were categorised by frequency of occurrence and reported alongside the number of participants affected in each study arm, as described in the following sections. Other trial characteristics, such as phase, sponsorship, and specific dates, including trial start and completion dates, were also extracted, tabulated, and analysed. This approach ensured consistent classification and comparability of AEs across trials.

### 2.4. Primary and Secondary Outcomes

The primary outcome was the safety profile of each drug subgroup, evaluated based on the number of participants who experienced OAEs, the specific types of OAEs that occurred significantly more frequently in the studied drug group compared to placebo, and the relative increase in risk associated with these events. Secondary outcomes included the number of participants experiencing OAEs at higher doses of the studied drug, the types of OAEs reported in high-dose trials, the number of participants with SAEs, the total number of reported OAEs and SAEs, the number of enrolled participants and those who discontinued due to AEs, as well as trial characteristics such as phase, interventional model, masking, sponsorship, and trial duration, with the latter reported as the median elapsed time in months.

In trials with an active control, “higher dose” was defined as any dose exceeding that of the studied intervention. Due to variability in dosing regimens across trials and lack of standardised dose thresholds, a relative within-trial comparison approach was adopted to ensure consistency.

### 2.5. Statistical Analysis

Percentages and medians with 95% confidence intervals (CIs) were reported. The elapsed time from the start to the completion of a trial was considered non-parametric and categorised based on the median value: values less than or equal to the median were dichotomised as 0, while values greater than the median were classified as 1. These values were compared using the chi-square test. We first filtered the extracted OAEs based on the number of affected participants. We selected only those that affected more than 0.1% of participants in the studied drug group or the active control group, where the studied drug was used at a higher dose. We then compared the proportions of these OAEs with those reported by participants who received a placebo in the corresponding trials using a Z-test for proportions. If an OAE was present in one group but absent in the other observed group, Fisher’s exact test was conducted instead of the Z-test for proportions to assess the relationship between proportions. We then conducted a meta-analysis of the filtered OAEs based on the previously subgrouped trials. Statistical heterogeneity across subgrouped trials was assessed using the I^2^ statistic, specifically evaluating variability in the number of participants affected by individual OAEs. An I^2^ value greater than 25% was considered indicative of substantial heterogeneity, reflecting a conservative threshold to account for variability across trials in the context of meta-epidemiological analyses, consistent with commonly used interpretations of I^2^ values in the literature [21]. For each AE, the choice of meta-analysis model was determined by the level of heterogeneity: a fixed-effects model was used when heterogeneity was low (I^2^ ≤ 25%), and a random-effects model (using the DerSimonian–Laird method) was used otherwise. The type of model applied (fixed or random effects) and the number of included studies are explicitly reported for each analysis in the results and/or figure legends. Meta-regression analyses were performed to explore potential sources of heterogeneity for each subgroup and filtered OAE, assessing the effects of participants’ mean age, sex, and treatment duration. Racial and ethnic data were excluded due to inconsistent reporting and predominance of White participants, and dosing frequency was excluded due to limited variability. Detailed regression coefficients, 95% CIs, and *p*-values are provided in Supplementary Table S3 with full datasets available in the OSF repository. No significant associations were identified unless otherwise stated.

In the next filtering step, based on the meta-analysis results, we ensured that both the RR value and the lower bound of the 95% CI were greater than 1 to identify OAEs that were truly more frequent than in the placebo group. The OAEs filtered in this way were presented based on their frequency of occurrence, categorised as affecting ≥10%, ≥1%, or ≥0.1% of participants, following the classification proposed by the Council for International Organizations of Medical Sciences (CIOMS) Working Group III, which defines AE frequencies as very common (≥10%), common (≥1% to <10%), and uncommon (≥0.1% to <1%) [22]. While this filtering step ensured that we focused on OAEs with the strongest and most statistically robust associations, it also meant that OAEs with elevated RR values but wide 95% CIs were excluded from the pooled results. These events, although not statistically significant, were documented during extraction and may still carry clinical relevance, particularly in subgroups with limited sample sizes.

Additionally, OAEs were presented according to MedDRA SOCs, along with the corresponding forest plots. A meta-analysis was conducted only for OAEs because they were reported in sufficient quantity and consistency across the trials. In contrast, SAEs were predominantly reported as absent (i.e., zero events), and their definitions and classifications varied considerably across studies, which limited the possibility of meaningful statistical pooling. All analyses were conducted using a two-tailed significance level of 0.05. Given that the included studies were RCTs with available incidence data, the RR was prioritised for graphic presentation and discussion interpretation, as it offers a more direct measure of increased risk and is more clinically intuitive. These analyses were performed using Microsoft Excel version 16.95.4 and R software, version 4.4.3 (R Foundation for Statistical Computing, Vienna, Austria). Differences were considered significant if *p* < 0.05. A formal risk of bias assessment was not conducted, as this study is meta-epidemiological in design and relies on registry-level summary data from ClinicalTrials.gov, where detailed methodological information required for such assessments is not consistently available.

## 3. Results

### 3.1. General Characteristics

**Figure 1:**
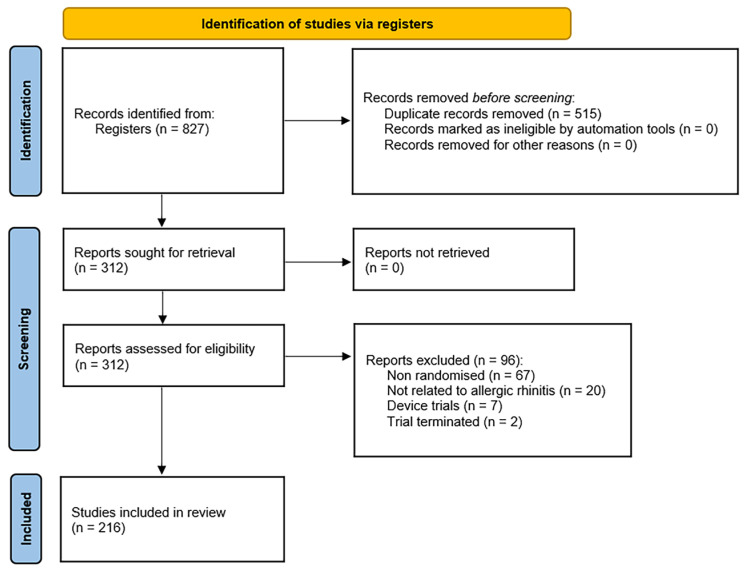
PRISMA 2020 flow diagram of study selection from ClinicalTrials.gov registry, adapted from Page et al. (2021) [23].

A total of 216 trials were analysed (Figure 1). Most trials were Phase 3 (48.15%), followed by Phase 4 (26.39%) and Phase 2 (16.67%). The most commonly used designs were parallel (83.33%) and crossover (14.81%). Quadruple (35.19%) and double blinding (32.41%) were the most common masking approaches. Table 1 presents the structure of the analysed trials based on the drugs they studied, including some basic characteristics. 175 (81.02%) trials were exclusively industry-sponsored, and 13 (6.02%) were secondarily industry-sponsored. Twenty-seven (12.50%) trials were non-industry-sponsored, and the sponsor was not specified for one (0.46%) trial.

The median elapsed time from trial completion to publication of results in the ClinicalTrials.gov registry was 21.7 months (95% CI 17.77–26.43), which is significantly longer than the trial duration itself, with a median of 10.3 months (95% CI 8.13–12.93). Industry-sponsored trials had a significantly shorter duration compared to the rest of the trials (χ^2^ = 18.72, *p* < 0.001) with a median of 5.1 months (95% CI 3.07–7.17).

The analysed RCTs for AR treatments enrolled 88,453 participants. 1684 (1.90%) participants discontinued due to AEs. 587 (0.66%) participants reported SAEs, while 19,591 (22.15%) observed OAEs. An increased risk of SAE occurrence was observed only with SCIT, with an RR of 1.85 (95% CI 1.1–3.14) and an OR of 1.91 (95% CI 1.11–3.29). As no heterogeneity was detected (I^2^ = 0.0%, *p* = 0.965), a fixed-effects model was applied.

Table 2 presents the number of participants affected by AEs and the reported AEs per trial.

Furthermore, Table 3 presents the number of participants affected by OAEs and the reported OAEs by the studied drug, active control, and placebo groups.

### 3.2. IN Drugs

Most of the analysed trials (N = 120 [55.56%]) studied the efficacy and safety of IN drugs exclusively, enrolling 51,877 participants (Table 1). This group’s median duration of trials is 5 months (95% CI 7.76–11.40). Trials investigating INCS and INAH, either alone or in combination, account for 99.42% of the participants in this group.

#### 3.2.1. INCS

**Figure 2:**
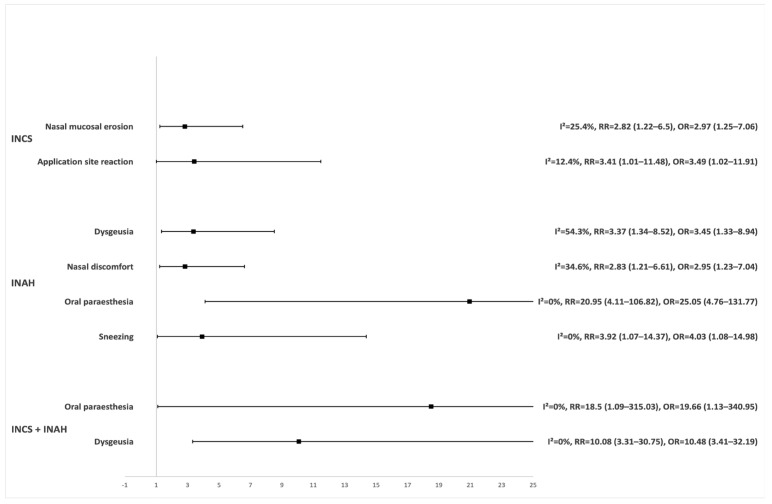
Relative risk (RR) with 95% confidence intervals for OAEs significantly more frequent with INCS, INAH, and their combination compared to the placebo group.

Most trials in this group (N = 79 [65.83%]) examined the efficacy and safety of INCS, either alone or combined with other, non-INAH intranasal drugs. This subgroup also included the majority of participants in the entire IN group (N = 28,184 [54.33%]). Within this subgroup, 11,851 participants received the studied INCS and were analysed, of which 1950 (16.45%) experienced an OAE and 128 (1.08%) discontinued due to AEs. There was no evidence of heterogeneity among the trials in reported affected participants by OAEs (I^2^ = 0.0%, *p* = 0.835). Furthermore, the overall risk of OAEs in the studied drug group from INCS monotherapy was not significantly higher compared to placebo (RR = 1.05 [95% CI 1.00–1.11], OR = 1.08 [95% CI 1.00–1.18]). A separate analysis of individual OAEs revealed a significant increase in the incidence of affected participants and the risk of nasal mucosal erosion and application site reaction compared to the placebo (Figure 2 and Table 4).

Within the INCS subgroup, 2794 participants received the studied INCS at higher doses as an active control, of which 650 (23.26%) experienced an OAE and 41 (1.47%) discontinued due to AEs. There was no evidence of heterogeneity among the trials in reported affected participants by OAEs (I^2^ = 0.0%, *p* = 0.515). The overall risk of OAEs in the active control group, where participants received a higher dose of INCS, was not significantly higher than placebo (RR = 1.06 [95% CI 0.96–1.17], OR = 1.09 [95% CI 0.92–1.28]). A potential increase in the risk of urinary tract infections (UTIs) was observed in participants receiving higher doses of INCS compared to placebo (Supplementary Table S1). A longer treatment duration showed a statistically significant but clinically negligible association with this outcome (RR = 1.02 [95% CI 1.00–1.04], *p* = 0.044).

#### 3.2.2. INAH

Twenty-two (18.33%) trials in this group focused on the efficacy and safety of INAH, involving 12,275 (23.66%) participants from the entire group. Within this subgroup, 4528 participants received the studied INAH, of which 1333 (29.44%) were affected by an OAE and 173 (3.82%) discontinued due to AEs. There was considerable heterogeneity among the INAH trials in reporting affected participants by OAEs (I^2^ = 68.7%, *p* < 0.001). The risk was higher in the studied INAH group; however, the increase was not statistically significant based on the RR of 1.33 (95% CI 0.99–1.80), whereas the OR of 1.54 (95% CI 1.06–2.25) indicated a statistically significant association. Analysis of individual OAEs revealed a significantly higher risk of dysgeusia, nasal discomfort, oral paraesthesia and sneezing compared to the placebo group (Figure 2 and Table 4).

Within the INAH subgroup, 1886 participants received the studied INAH at higher doses as an active control, of which 261 (13.84%) experienced an OAE and 14 (0.74%) discontinued due to AEs. No statistically significant OAE increase was observed with higher doses of INAH (RR = 1.45 [95% CI 0.84–2.50], OR = 1.57 [95% CI 0.85–2.90]), while considerable heterogeneity was present among the trials (I^2^ = 62.4%, *p* = 0.006). However, further analysis of each OAE associated with higher dose INAH revealed a notably increased incidence of dysgeusia compared to placebo (Supplementary Table S1), with a higher mean age of participants slightly further increasing the risk (RR = 1.07 [95% CI 1.00–1.15], *p* = 0.048).

#### 3.2.3. Combination of INCS and INAH

The combination of INCS and INAH was studied in 15 (12.50%) trials, involving 11,117 (21.43%) participants from the entire group. Within this subgroup, 3827 participants received the studied combination of INCS and INAH, of which 334 (8.73%) were affected by an OAE and 28 (0.73%) discontinued due to AEs. Analysis of OAE incidence indicated an increased risk associated with this combination (RR = 1.58 [95% CI 1.08–2.30], OR = 1.64 [95% CI 1.09–2.47]), supported by significant heterogeneity across these trials (I^2^ = 42.9%, *p* = 0.044). Longer treatment duration within this combination was associated with a statistically significant but negligible change in risk of OAEs (RR = 1.00 [95% CI 1.00–1.00], *p* = 0.002). The combination therapy was associated with elevated risks of dysgeusia and oral paraesthesia (Figure 2 and Table 4), with a higher mean age of participants slightly further increasing the risk of oral paraesthesia (RR = 1.09 [95% CI 1.00–1.18], *p* = 0.041). No trials used a higher dose of the studied drug used as the active control.

### 3.3. AIT

The second most represented group of trials (N = 49 [22.69%]) studied the efficacy and safety of AIT, enrolling 18,015 participants. These trials, with a median duration of 18.5 months (18.73–29.80), were significantly longer than the other trials (χ^2^ = 43.395, *p* < 0.001). Trials on the efficacy and safety of SLIT and SCIT accounted for 99.52% of the participants in this group. The remainder was related to trials on ILIT.

#### 3.3.1. SLIT

**Figure 3:**
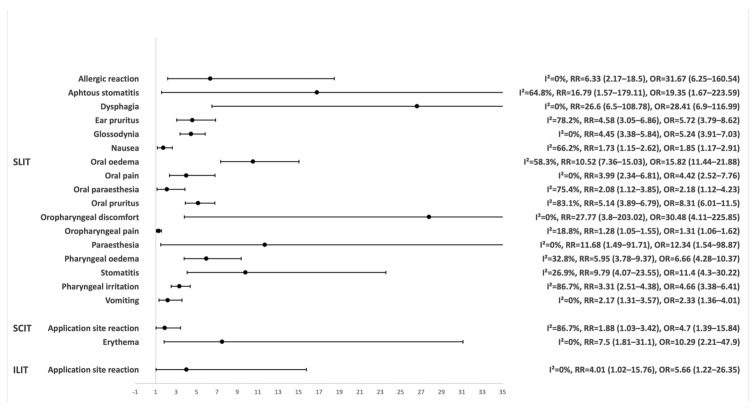
Relative risk (RR) with 95% confidence intervals for OAEs significantly more frequent with SLIT and ILIT compared to the placebo group.

Most trials in this group (N = 31 [63.27%]) focused on the safety and efficacy of SLIT. This subgroup included most participants in the entire group (N = 14,147 [78.53%]). Within this subgroup, 6369 participants received the studied SLIT, of which 4137 (64.96%) were affected by an OAE and 372 (5.84%) discontinued due to AEs. Meta-analysis revealed an increased risk of OAEs associated with SLIT (RR = 1.31 [95% CI 1.20–1.44], OR = 2.12 [95% CI 1.76–2.56]), supported by significant heterogeneity across these trials (I^2^ = 87.8%, *p* < 0.001). According to our results, the highest risk was observed for oropharyngeal discomfort and dysphagia, and numerous other, predominantly gastrointestinal and respiratory OAEs presented in Figure 3 and Table 5. Furthermore, a longer treatment duration slightly increased the risk of aphthous stomatitis (RR = 1.01 [95% CI 1.00–1.01], *p* = 0.046) and nausea (RR = 1.01 [95% CI 1.00–1.01], *p* = 0.004), while a higher mean age of participants was associated with a marginally reduced risk of oral pain (RR = 0.95 [95% CI 0.91–0.99], *p* = 0.013).

Within the SLIT subgroup, 1264 participants received the studied SLIT at higher doses as an active control, of which 758 (59.97%) experienced an OAE and 96 (7.59%) discontinued due to AEs. The analysis demonstrated an increased risk of OAEs associated with a higher dose of SLIT (RR = 1.39 [95% CI 1.13–1.71], OR = 2.21 [95% CI 1.60–3.06]), supported by significant heterogeneity across these trials (I^2^ = 67.4%, *p* = 0.003). A higher mean age of participants slightly increased the overall risk of OAEs (RR = 1.03 [95% CI 1.02–1.05], *p* < 0.001), while a higher proportion of female participants was associated with a decreased risk (RR = 0.39 [95% CI 0.17–0.89], *p* = 0.025). Additionally, longer treatment duration had a negligible but statistically significant effect (RR = 1.00 [95% CI 1.00–1.00], *p* = 0.034). In the higher dose SLIT group, oral and ear pruritus, oropharyngeal oedema and pharyngeal irritation were more frequent compared to placebo. A dose-dependent increase was observed for pharyngeal oedema, ear and oral pruritus compared to the regular dose (Supplementary Table S2). Furthermore, a higher mean age of participants slightly increased the risk of oral pruritus (RR = 1.06 [95% CI 1.00–1.11], *p* = 0.037), whereas longer treatment duration was associated with a negligible but statistically significant increase in the risk of throat irritation (RR = 1.00 [95% CI 1.00–1.01], *p* = 0.046).

#### 3.3.2. SCIT

Another significant subgroup included trials on the efficacy and safety of SCIT. These trials involved 3781 (20.99%) participants. Within this subgroup, 1330 participants received the studied SCIT, of which 718 (53.98%) were affected by an OAE and 15 (1.13%) discontinued due to AEs. No significant OAE increase was detected in the risk of OAEs associated with the use of SCIT (RR = 1.08 [95% CI 0.91–1.27], OR = 1.29 [95% CI 0.78–2.16]), despite considerable heterogeneity among the trials (I^2^ = 58.3%, *p* = 0.004). However, in the separate meta-analysis of each OAE related to SCIT, a significantly increased incidence of application site reactions and erythema was observed compared to placebo (Figure 3 and Table 5).

Only 2 (14.29%) trials in this subgroup used a higher dose of SCIT as the active control. As in a regular dose, the meta-analysis indicated no statistically significant increase in the risk of OAEs with the use of a higher dose of SCIT (RR = 1.13 [95% CI 0.05–23.38], OR = 1.23 [95% CI 0.02–75.32]), while heterogeneity was present among the trials (I^2^ = 35.5%, *p* = 0.213). No OAE from this group was associated with an increased risk relative to placebo.

#### 3.3.3. ILIT

In the subgroup of ILIT trials, which involved 87 (0.48%) participants, none of the enrolled participants discontinued due to AEs. Within this subgroup, 40 participants received the studied ILIT, of which 25 (62.50%) were affected by an OAE. Meta-analysis of reported affected participants by OAEs from these trials indicates an increased risk of OAEs associated with ILIT (RR = 2.51 [95% CI 1.31–4.80], OR = 7.35 [95% CI 1.92–28.11]), without heterogeneity across these trials (I^2^ = 0.0%, *p* = 0.404). Furthermore, an additional meta-analysis of each reported OAE demonstrated a statistically significant increase in the risk of application site reactions associated with ILIT (Figure 3 and Table 5). A higher dosage of ILIT was used as the active control in no trial.

### 3.4. Oral Drugs

The third most represented group of studies (N = 39 [18.06%]) studied the efficacy and safety of oral drugs for the treatment of AR. These studies had the shortest duration, with a median duration of 4 months (95% CI 5.72–14.85). The group included a total of 17,626 participants. Studies examining OAH and LTRA, either alone or in combination, encompassed 15,613 (88.58%) participants in this group.

#### 3.4.1. OAH

Most studies in this group (N = 15 [51.72%]) focused on the efficacy and safety of OAH. This subgroup encompassed a smaller portion of the entire group (N = 5107 [29.33%]). Within this subgroup, 2446 participants received the studied OAH, of which 237 (9.69%) were affected by OAE and 23 (0.94%) discontinued due to AEs. The meta-analysis of participants affected by OAEs indicated no statistically significant increase in the risk of OAEs with the use of OAH (RR = 1.01 [95% CI 0.86–1.20], OR = 1.07 [95% CI 0.86–1.34]), and without heterogeneity among the trials (I^2^ = 0.0%, *p* = 0.998). However, an additional analysis of each reported OAE demonstrated a statistically significant increase in the risk of Upper respiratory tract infections (URTIs) associated with use of OAH compared to placebo (I^2^ = 16.3%, RR = 3.1 [95% CI 1.73-5.55], OR = 3.84 [95% CI 1.99–7.42]) (Table 6), with longer treatment duration slightly further increasing this risk (RR = 1.01 [95% CI 1.00–1.02], *p* = 0.021).

Only one (6.67%) trial used the studied OAH as the active control in a higher dose. The RR of 1.88 (95% CI 0.86–4.12) and OR of 1.96 (95% CI 0.85–4.51) from that trial did not demonstrate a significantly increased risk of OAEs compared to placebo.

#### 3.4.2. LTRA

The second subgroup consists of 10 (34.48%) trials on LTRA, involving 7436 (42.19%) participants. Within this subgroup, 3587 participants received the studied LTRA, of which 291 (8.11%) were affected by OAEs and 95 (2.65%) discontinued due to AEs. The meta-analysis of participants affected by OAEs indicated no statistically significant increase in the risk of OAEs with the use of LTRA (RR = 1.06 [95% CI 0.87–1.29], OR = 1.07 [95% CI 0.85–1.34]), and without heterogeneity among the trials (I^2^ = 0.0%, *p* = 0.703). No trial administered a higher dosage of LTRA as the active control.

#### 3.4.3. Combination of OAH and LTRA

The combination of OAH and LTRA encompasses 4 (10.26%) studies with 3007 (17.06%) participants. Within this subgroup, 767 participants received the studied combination, of which 21 (2.74%) were affected by OAEs and 5 (0.65%) discontinued due to AEs. The meta-analysis of participants affected by OAEs indicated no statistically significant increase in the risk of OAEs with the use of the combination (RR = 0.72 [95% CI 0.31–1.69], OR = 0.72 [95% CI 0.30–1.74]), and without heterogeneity among the trials (I^2^ = 0.0%, *p* = 0.603). No trial used the combination at a higher dose as the active control.

## 4. Discussion

This meta-epidemiological study aimed to identify patterns in AE reporting across RCTs rather than establish causal relationships or precise risk estimates for individual interventions. Our analysis showed that intranasal drug trials dominate the landscape, particularly INCS, which remain the recommended first-line therapy for AR [24]. In contrast, AIT trials were significantly longer, reflecting the need to evaluate long-term efficacy and immunological tolerance, while oral drug trials were shorter in duration [25]. Due to the limited number of trials involving some drug classes, certain subgroup analyses were not feasible and were therefore excluded.

The number of reported OAEs exceeded the number of affected participants across IN, AIT, and oral drug groups (Table 3), reflecting the occurrence of multiple events per participant. The table also indicates a higher rate of affected participants by OAEs in the active control group compared to the studied drug group for trials where a higher dose of the studied drug was used as the active control.

Although numerous studies on the safety of IN, AIT, and oral drugs report potential OAEs, we have selected only those OAEs that were significantly more frequent in both the studied drug group and the higher-dose group compared to the placebo group (Table 4, Table 5 and Table 6 and Supplementary Tables S1 and S2). AIT trials deviate in the number of affected participants compared to IN and oral drug trials, which exhibit similar proportions, consistent with the results of a recent study by Torres et al. [26].

### 4.1. IN Drugs

INCS showed a generally favourable safety profile compared to INAH, consistent with prior studies [7]. Combination therapy also appeared safe and may enhance efficacy in moderate to severe AR. INAH was linked with taste disturbance and nasal discomfort, highlighting the importance of careful selection in clinical practice [4]. Table 4 indicates a favourable safety profile for INCS, with fewer participants affected by OAEs than INAH, which is also a less effective drug in controlling AR symptoms than INCS [4]. Although epistaxis is frequently reported as one of the most common AEs associated with INCS use, in our study, it was also the most frequently reported local OAE, not only in the studied INCS group but also within the placebo group. Consequently, the meta-analysis did not confirm an increased risk of epistaxis associated with INCS use. This discrepancy may partially reflect differences in the risk profiles of individual INCS formulations. For instance, a systematic review and meta-analysis by Wu et al. demonstrated an overall relative risk of epistaxis of 1.48 (95% CI, 1.32–1.67) for all INCSs compared to placebo, with certain formulations like beclomethasone hydrofluoroalkane and fluticasone furoate associated with higher risks, while others like beclomethasone aqueous and ciclesonide aqueous were associated with lower risks [27]. Therefore, the variability in epistaxis risk among different INCS formulations may contribute to the observed discrepancies in our study. Our results indicate that nasal mucosal erosion is the most significant risk associated with INCS. However, this AE was uncommonly reported overall in the studied group. The moderately wide 95% CI indicates some uncertainty in the risk estimate, reflecting some uncertainty in the precise risk assessment and underscoring the potential need for larger sample sizes or additional studies to enhance the accuracy of the estimate. Application site reactions demonstrated a moderately increased and borderline statistically significant risk. However, the wide 95% CI indicates substantial uncertainty. The term may have been used inconsistently across studies, potentially including epistaxis, mucosal erosion, or general discomfort, which limits its specificity. The findings of a study from the Netherlands align with our observations, suggesting that although application site reactions are reported, their interpretation requires careful consideration of reporting practices and administration techniques [28].

Using INAH carries an increased risk of dysgeusia and nasal discomfort, which are commonly reported OAEs from this group. The results are consistent with other studies that find a better safety profile for INCS and identify dysgeusia as exclusively associated with INAH [7]. This may be explained by the direct deposition of INAH on the oropharyngeal mucosa and subsequent stimulation of taste receptors, which is a well-recognized mechanism underlying dysgeusia [14]. Oral paraesthesia and sneezing were both associated with a statistically significant risk increase for the group using INAH compared to the placebo group. Oral paraesthesia showed a markedly elevated relative risk, while sneezing demonstrated a more moderate increase. The findings of previous studies [26,29] align with our observations, where oral paraesthesia and sneezing were associated with a statistically significant increase in risk among INAH users. However, both estimates were accompanied by wide 95% CIs, reflecting uncertainty in the precise quantification of risk. This concordance highlights the need for standardised reporting and further research to better understand the safety profile of INAH. Furthermore, the combination of INCS and INAH was associated with an even greater risk of dysgeusia, with older age slightly contributing to the risk of oral paraesthesia in this group, suggesting careful monitoring in older patients.

A potential increase in UTIs was observed in the higher-dose INCS group. However, this finding was based on a limited number of events and imprecise estimates, with wide CIs indicating substantial uncertainty. The underlying mechanism remains unclear, and this observation should be interpreted with caution and considered hypothesis-generating rather than confirmatory.

In contrast, the administration of a higher dose of INAH was associated with a significantly increased risk of dysgeusia compared to the regular dose, suggesting a potential dose-dependent relationship with older age of participants slightly further contributing to this risk (Supplementary Table S1).

### 4.2. AIT

Within AIT, SLIT was associated with a broad spectrum of local OAEs, particularly oral and oropharyngeal reactions [8,30]. These local reactions are likely related to mucosal exposure to allergens and activation of local immune responses, including IgE-mediated pathways and inflammatory mediator release at the site of administration [REF1]. While SCIT showed higher rates of SAEs despite fewer overall OAEs, both overall and within the studied drug group, suggesting a less favourable safety profile compared to SLIT. This aligns with findings from a recent large-scale comparison of AIT modalities [10]. Additionally, a significantly increased risk of SAE occurrence was observed exclusively with SCIT, highlighting its distinct safety profile compared to other interventions. However, despite its poorer safety profile, SCIT is more effective than SLIT [31]. ILIT remains understudied but indicated an elevated risk of local reactions, accompanied by a wide 95% CI, indicating the necessity for a larger sample size and an improved design of future safety analyses. Very common OAEs associated with SLIT (see Table 5) are primarily local and showed a significantly increased risk compared to placebo (Figure 3), underscoring the substantial local impact of SLIT. Systemic reactions were also more frequent than with placebo, though to a lesser extent than local events, with notably wide 95% CIs reflecting substantial uncertainty in the corresponding risk estimates and highlighting the need for further investigation. Moreover, the slight increase in risk of aphthous stomatitis and nausea with longer treatment duration is unlikely to be clinically meaningful, while the marginally lower risk of oral pain in older participants suggests age may modestly influence local tolerability. These findings should be interpreted in the context of event frequency, data quality, and clinical relevance. SLIT is associated with a broad spectrum of both common and uncommon OAEs, as illustrated in Table 5, aligning with the observations of other researchers [9,32]. In contrast, SCIT has a much lower range of OAEs, mainly skin-related, with application site reactions being the only very common OAE. Pruritus and erythema are classified as common, which is also consistent with a recent study [9].

Overall, our meta-analysis did not reveal a statistically significant increase in the risk of any OAEs with SCIT compared to placebo, indicating a favourable safety profile in this regard. In contrast, ILIT was associated with a significantly elevated risk of application site reaction, consistent with findings reported in previous studies [33]. Notably, higher-dose SLIT was associated with a significantly increased incidence of local OAEs, particularly oral oedema, oral and ear pruritus, and throat irritation, compared to placebo. This dose-dependent trend, especially the elevated rates of oral and ear pruritus compared to regular-dose SLIT, aligns with earlier observations suggesting that local reactions intensify with increased allergen exposure [34]. Meta-regression results indicate that older age exerts a measurable but minimal influence on the overall risk of OAEs and oral pruritus, suggesting slightly increased mucosal sensitivity in older participants. The lower risk observed in females may reflect sex-related differences in immune responsiveness or symptom reporting, though the effect size is small and likely of limited clinical relevance. The effect of treatment duration, while statistically significant, appears negligible in practice, supporting the stability of the OAE profile over time. Importantly, no significant increase in the risk of systemic or local OAEs was observed with standard-dose SCIT or ILIT in comparison to placebo, supporting their relative tolerability.

### 4.3. Oral Drugs

OAH remain a mainstay in AR management and demonstrated an overall favourable safety profile [11]. Furthermore, second-generation oral antihistamines remain key monotherapies for treating seasonal AR due to their efficacy, safety, and patient preference [4,11]. OAH was the most frequently studied but encompassed a smaller proportion of the total participants (29.33%). LTRA trials showed higher discontinuation rates but no consistent increase in AEs. URTIs were a common OAE for both drugs, but a significant increase compared to placebo occurred only with OAH. Longer treatment duration slightly increased this risk, though the effect was minimal and unlikely to be clinically relevant. This increase in URTIs risk may be related to the drying effect of antihistamines on mucosal surfaces, which can impair the respiratory tract’s natural defence mechanisms and potentially alter immune responses [35]. However, a meta-analysis from Xu et al. found similar efficacy and safety profiles for these two drugs [36]. The combination of OAH and LTRA appeared safe, although evidence remains limited [37].

Overall, a cross-modal comparison of safety profiles across treatment groups reveals important differences in tolerability. IN drugs, particularly INCS, demonstrate the most favourable safety profile, with no significant increase in overall OAEs compared to placebo and only a limited number of primarily local AEs. INAH and combination therapies show a higher frequency of local reactions, though these are generally mild. In contrast, AIT is associated with a broader and more frequent spectrum of OAEs, particularly with SLIT, where local oropharyngeal reactions predominate. SCIT, while associated with fewer OAEs overall, carries a higher risk of SAEs, distinguishing it from other treatment modalities. Oral drugs generally exhibit a favourable safety profile, with minimal increases in OAEs and relatively low discontinuation rates, particularly when used in combination. These findings highlight clinically relevant differences in safety across treatment modalities and support individualized treatment selection based on both efficacy and tolerability. Importantly, most of the observed OAEs were mild and localized, suggesting that while statistically significant differences exist between treatments, their clinical impact is often limited. Therefore, treatment decisions should consider not only the frequency of OAEs but also their severity and overall tolerability.

Our findings may also provide additional context for current guideline recommendations, such as those from ARIA and EAACI, which emphasize the use of INCS as first-line therapy for AR. The favourable safety profile observed in our analysis further supports these recommendations, while the distinct safety patterns identified for AIT and oral drugs highlight the importance of individualized risk–benefit assessment. Although our results are not intended to inform guideline development directly, they may contribute to a more nuanced understanding of treatment tolerability and support future updates that integrate both efficacy and safety considerations.

### 4.4. Study Limitations

We analysed trials only registered on ClinicalTrials.gov. This registry-based design inherently limits the level of detail available for analysis, as it relies on summary-level data rather than full reports or individual participant data. Currently, 17 other primary registries in the World Health Organization (WHO) registry network meet the requirements of the International Committee of Medical Journal Editors (ICMJE) [38]. As a result, there may be some gaps or limitations in the completeness and accuracy of the data. However, ClinicalTrials.gov is the largest clinical trial registry [39] offering a robust and reliable dataset for analysis. Although some of the drugs examined in this study had fewer trials or participants, we have taken care to interpret these findings with appropriate caution, ensuring the strength and validity of our conclusions. Moreover, despite our strategy of including all reported OAEs irrespective of their presumed causality to minimise reporting bias, variability in how individual trials defined and reported OAEs may still contribute to residual heterogeneity. In addition, inconsistencies in OAE reporting across the original trials may have influenced the observed outcomes. Some trials provided more general OAE categories, while others reported more specific events, potentially leading to ambiguity. Additionally, selective reporting of only TRAEs in some trials could have resulted in underreporting. These factors should be considered when interpreting the findings and their generalizability. Also, a formal risk of bias assessment was not conducted, as this study relied exclusively on registry-level summary data from ClinicalTrials.gov, where detailed methodological information required for such evaluations is often unavailable or inconsistently reported. Given the meta-epidemiological design, the focus was on identifying aggregate patterns in AE reporting rather than assessing the internal validity of individual trials. Another limitation concerns the exclusion of racial and ethnic variables, as well as dosing frequency, from the meta-regression due to insufficient variability and inconsistent reporting across trials, which may have limited the exploration of potential sources of heterogeneity. Furthermore, although meta-regression was performed for age, sex, and treatment duration and found no significant effects, the overall heterogeneity observed across INAH, higher doses INAH, INCS + INAH combination, SLIT, higher doses SLIT, SCIT, and higher doses SCIT trials (before filtering for individual OAEs) may instead reflect other unmeasured factors, such as differences in trial design, drug formulations, reporting practices, or participant populations. 

It is important to note that several OAEs demonstrated increased RR values but did not achieve statistical significance. These findings were often associated with limited event numbers, small sample sizes, or high variability across studies. While they were excluded from the main meta-analysis results due to our predefined filtering criteria, their potential clinical significance should not be overlooked. These events warrant further investigation in future studies with larger cohorts or pooled individual participant data to better clarify their true association with treatment exposure.

In addition, emerging therapeutic approaches for allergic rhinitis, including herbal medicines, nasal saline irrigation, and biological therapies, have gained increasing attention in recent years. However, these interventions were not systematically included in our analysis due to the predefined focus on guideline-recommended pharmacological treatments and the limited availability of adequately reported RCT data within ClinicalTrials.gov. Future studies should further explore the safety profiles of these therapies, particularly as their role in clinical practice continues to evolve.

## 5. Conclusions

Our meta-epidemiological analysis of completed RCTs on AR treatments highlights important differences in safety profiles across drug classes. INCS demonstrated the most favourable balance of efficacy and safety, confirming their role as first-line therapy. AIT showed distinct safety patterns, with SLIT associated with frequent local reactions and SCIT with a higher risk of SAEs, while ILIT remains insufficiently studied. Oral therapies were generally well tolerated, with only minor safety concerns.

Most OAEs were mild and localized, suggesting limited clinical impact despite observed differences between treatments.

Overall, our findings reinforce the central role of INCS, the need for individualised risk–benefit assessment when considering AIT, and the value of continued research to clarify long-term safety outcomes.

## Figures and Tables

**Table 1:** Pharmacological treatments for allergic rhinitis evaluated in ClinicalTrials.gov-registered trials.

Drug Group	Drug Subgroup	Drugs	N (%)	Mean Age (Range, Years)	Female (Range, %)	Treatment Duration (Range, Days)
IN	INCS	INCS	74 (34.26)	6.12–53.00	4.55–81.65	1–728
		INCS + INDEC	2 (0.93)	28.60–28.60	64.06–66.20	14–28
		INCS + B2 agonist	1 (0.46)	20.40–20.40	31.25–31.25	NA
	INAH	INAH	21 (9.72)	3.37–44.30	14.67–68.99	7–365
		INAH + INAH	1 (0.46)	NA	72.55–72.55	1–1
	INCS + INAH	INCS + INAH	15 (6.94)	8.10–43.64	17.22–67.39	1–365
	OTHER	INDEC	1 (0.46)	NA	57.14–57.14	2–2
		Triamcinolone acetonid	2 (0.93)	23.60–23.60	64.29–64.29	7–7
		TLR 7 agonist	1 (0.46)	29.70–29.70	12.05–12.05	30–30
		Topical nasal spray	1 (0.46)	44.60–44.60	45.83–45.83	14–14
		Homeopathic drug	1 (0.46)	39.50–39.50	48.04–48.04	180–180
AIT	SLIT	SLIT	31 (14.35)	10.50–56.40	29.21–80.00	28–365
	SCIT	SCIT	14 (6.48)	12.10–39.60	31.71–70.97	6–365
		SCIT + dupilumab	1 (0.46)	33.00–33.00	48.54–48.54	112–112
	ILIT	ILIT	3 (1.39)	19.54–34.00	33.33–57.14	90–365
Oral	OAH	OAH	15 (6.94)	3.78–44.20	0.00–80.00	1–90
	LTRA	LTRA	10 (4.63)	5.40–36.30	0.00–68.52	2–365
	OAH + LTRA	OAH + LTRA	4 (1.85)	29.70–37.60	53.01–64.72	14–28
	ODEC	ODEC	5 (2.31)	27.05–40.10	58.97–66.04	1–7
	OTHER	Lipoic acid	1 (0.46)	40.90–40.90	56.36–56.36	60–60
		Broccoli sprouts	1 (0.46)	33.10–33.10	47.06–47.06	3–3
		Probiotic mixture	2 (0.93)	26.00–35.50	60.00–68.79	56–60
		Herbal formula	1 (0.46)	41.19–41.19	65.87–65.87	28–28
Ocular	OcAH	OcAH	2 (0.93)	NA	70.00–85.71	1–10
Comb.	Intranasal and oral	INCS + OAH	1 (0.46)	25.60–25.60	49.72–49.72	15–15
		INCS + Broccoli sprouts	1 (0.46)	NA	25.53–25.53	21–21
		INCS + vitamin D3	1 (0.46)	27.30–27.30	54.29–54.29	14–14
		INAH + OAH	1 (0.46)	37.90–37.90	27.78–27.78	63–63
	Intranasal and ocular	INCS + OcAH	1 (0.46)	31.10–31.10	33.33–33.33	7–7
Total			216 (100.00)	3.37–56.40	0.00–85.71	1–728

Abbreviations: IN—Intranasal, AIT—Allergen immunotherapy, Comb.—Combinations, INCS—Intranasal corticosteroid, INAH—Intranasal antihistamine, SLIT—Sublingual Immunotherapy, SCIT—Subcutaneous Immunotherapy, ILIT—Intralymphatic Immunotherapy, OAH—Oral antihistamine, LTRA—Leukotriene receptor antagonist, OcAH—Ocular antihistamine, INDEC—Intranasal decongestant, TLR—Toll-like receptor, ODEC—Oral decongestant, NA—Not available.

**Table 2:** Median number of participants and adverse events per trial across drug subgroups.

Drug Subgroup	Median of Participants per Trial (95% CI)	Median of Discontinuation Due to AEs per Trial (95% CI)	Median of Participants with SAEs per Trial (95% CI)	Median of Participants with OAEs per Trial (95% CI)
INCS	325 (292.61–429.64)	1.5 (2.40–5.26)	0 (0.58–2.27)	19 (36.76–80.01)
INAH	498 (332.66–783.25)	0 (0.00–8.42)	0 (0.00–0.88)	54 (36.31–88.35)
INCS + INAH	607 (493.05–989.22)	0 (0.00–4.60)	1 (0.18–2.42)	12 (1.13–76.27)
SLIT	381 (297.57–615.14)	6 (8.68–29.90)	2 (1.23–10.59)	131 (99.38–329.54)
SCIT	195 (0.00–1002.65)	5.5 (0.00–12.15)	11 (0.27–25.40)	155 (0.00–655.43)
ILIT	30 (10.25–47.76)	0 (0.00–0.00)	0 (0.00–8.84)	13 (0.00–27.31)
OAH	336 (45.71–580.96)	1.5 (0.00–3.62)	0 (0.00–0.60)	22 (0.00–43.46)
LTRA	1146 (759.81–1483.69)	21.5 (0.00–65.15)	1.5 (0.00–5.44)	50.5 (0.00–179.47)
OAH + LTRA	680.5 (0.00–1782.95)	3 (0.00–16.15)	1 (0.00–1.55)	14.5 (0.00–44.19)

Abbreviations: CI—confidence interval, AE—Adverse event, SAE—Serious adverse event, OAE—Other adverse event, INCS—Intranasal corticosteroid, INAH—Intranasal antihistamine, SLIT—Sublingual Immunotherapy, SCIT—Subcutaneous Immunotherapy, ILIT—Intralymphatic Immunotherapy, OAH—Oral antihistamine, LTRA—Leukotriene receptor antagonist. For display purposes, any lower 95% CI values below 0 were truncated to 0 to reflect the non-negative nature of participant counts.

**Table 3:** Median number of participants with OAEs and reported OAEs per trial across treatment groups.

Drug Subgroup	Studied Drug	Active Control	Placebo
	Median of participants with OAEs per trial (95% CI)		
INCS	7 (15.15–35.88)	13 (13.21–45.27)	7 (13.12–29.15)
INAH	21 (11.00–27.67)	26 (15.11–37.77)	10 (3.39–29.73)
INCS + INAH	6 (0.08–26.52)	5 (0.88–38.32)	1 (0.00–11.93)
SLIT	36 (31.10–107.26)	69 (40.59–140.14)	26 (20.63–89.19)
SCIT	58.5 (0.00–206.63)	55.5 (0.00–247.68)	41 (0.00–208.43)
OAH	6.5 (0.00–15.86)	10 (0.00–19.75)	3.5 (0.00–8.54)
LTRA	20 (0.00–90.91)	10 (0.00–19.87)	20.5 (0.00–70.18)
	Median of reported OAEs per trial (95% CI)		
INCS	10 (22.36–62.04)	14 (15.40–83.36)	10 (19.86–55.73)
INAH	21 (0.00–68.41)	28 (0.00–126.51)	15 (0.00–70.31)
INCS + INAH	6.5 (0.22–26.58)	5 (0.77–39.23)	1.5 (0.00–12.00)
SLIT	106 (58.67–329.87)	260 (125.54–425.37)	75 (30.52–237.67)
SCIT	311.5 (69.50–587.84)	323 (94.69–505.64)	100.5 (0.00–405.03)
OAH	7.5 (0.00–16.16)	10 (0.00–19.75)	3.5 (0.00–8.96)
LTRA	20 (0.00–93.94)	10 (0.00–19.87)	20.5 (0.00–75.17)

Abbreviations: CI—confidence interval, OAE—Other adverse event, INCS—Intranasal corticosteroid, INAH—Intranasal antihistamine, SLIT—Sublingual Immunotherapy, SCIT—Subcutaneous Immunotherapy, OAH—Oral antihistamine, LTRA—Leukotriene receptor antagonist. For display purposes, any lower 95% CI values below 0 were truncated to 0 to reflect the non-negative nature of participant counts.

**Table 4:** Frequency of OAEs significantly more frequent with INCS, INAH, and their combination compared to the placebo group.

Organ System	Very Common OAE (≥10% of Affected Participants; *p* Value)	Common OAE (≥1% to <10% of Affected Participants; *p* Value)	Uncommon OAE (≥0.1% to <1% of Affected Participants; *p* Value)
INCS			
Respiratory, thoracic and mediastinal disorders	None	None	Nasal mucosal erosion (0.67%; <0.001)
General disorders and administration site conditions	None	None	Application site reaction (0.36%; <0.001)
INAH			
Gastrointestinal disorders	None	None	Oral paraesthesia (0.84%; <0.001)
Nervous system disorders	None	Dysgeusia (3.93%; <0.001)	None
Respiratory, thoracic and mediastinal disorders	None	Nasal discomfort (2.14%; <0.001)	Sneezing (0.68%; <0.001)
INCS + INAH			
Gastrointestinal disorders	None	None	Oral paraesthesia (0.24%; 0.003)
Nervous system disorders	None	Dysgeusia (1.25%; <0.001)	None

Abbreviations: OAE—Other adverse event, INCS—Intranasal corticosteroid, INAH—Intranasal antihistamine, None—no OAEs met the criteria for this cell.

**Table 5:** The most frequently reported OAEs significantly more frequent in the SLIT and SCIT groups compared to the placebo group.

Organ System	Very Common OAE (≥10% of Affected Participants; *p* Value)	Common OAE (≥1% to <10% of Affected Participants; *p* Value)	Uncommon OAE (≥0.1% to <1% of Affected Participants; *p* Value)
SLIT			
Ear and labyrinth disorders	Ear pruritus (16.77%; <0.001)		
Gastrointestinal disorders	Oral pruritus (32.96%; <0.001)	Nausea (5.45%; <0.001)	Dysphagia (0.83%; <0.001)
	Oral oedema (22.26%; <0.001)	Oral paraesthesia (4.41%; <0.001)	Vomiting (0.77%; 0.001)
	None	Glossodynia (4.21%; <0.001)	None
	None	Stomatitis (1.41%; <0.001)	None
	None	Oral pain (1.00%; <0.001)	None
Immune system disorders	None	None	Allergic reaction (0.30%; 0.001)
Infections and infestations	None	Aphtous stomatitis (2.04%; <0.001)	None
Nervous system disorders	None	None	Paraesthesia (0.20%; <0.001)
Respiratory, thoracic and mediastinal disorders	Pharyngeal irritation (29.85%; <0.001)	Pharyngeal oedema (4.93%; <0.001)	Oropharyngeal discomfort (0.41%; <0.001)
	None	Oropharyngeal pain (3.55%; 0.006)	None
SCIT			
General disorders and administration site conditions	Application site reaction (14.51%; <0.001)	None	None
Skin and subcutaneous tissue disorders	None	Erythema (1.13%; 0.002)	None
ILIT			
General disorders and administration site conditions	Application site reaction (32.50%; 0.001)	None	None

Abbreviations: OAE—Other adverse event, SLIT—Sublingual immunotherapy, ILIT—Intralymphatic immunotherapy, None—no OAEs met the criteria for this cell.

**Table 6:** The most common OAEs more frequent in the OAH group compared to the placebo group.

Organ System	Very Common OAE (≥10% of Affected Participants; *p* Value)	Common OAE (≥1% to <10% of Affected Participants; *p* Value)	Uncommon OAE (≥0.1% to <1% of Affected Participants; *p* Value)
OAH			
Infections and infestations	None	URTIs (2.70%; 0.022)	None

Abbreviations: OAE—Other adverse event, OAH—Oral antihistamine, URTIs—Upper respiratory tract infections, None—no OAEs met the criteria for this cell.

## Data Availability

This study was registered in the Open Science Framework (OSF) repository, DOI: https://doi.org/10.17605/OSF.IO/4J3GF. No formal protocol was prepared. The datasets generated and/or analysed during the current study are available at the same OSF repository.

## Supplementary Materials

The following supporting information can be downloaded at: https://www.mdpi.com/article/10.3390/pharmaceutics18050581/s1, Completed PRISMA checklist; Table S1: OAEs significantly more frequent in the high-dose INAH group compared to placebo; Table S2: OAEs significantly more frequent in the high-dose SLIT group compared to placebo; Table S3: Meta-regression results for factors associated with adverse events across treatment subgroups.

## Abbreviations

The following abbreviations are used in this manuscript:

AE	Adverse Event
AIT	Allergy Immunotherapy
AR	Allergic rhinitis
CIOMS	Council for International Organizations of Medical Sciences
CTCAE	Common Terminology Criteria for Adverse Events
ICMJE	International Committee of Medical Journal Editors
IN	Intranasal
INAH	Intranasal antihistamine
INCS	Intranasal Corticosteroid
LTRA	Leukotriene Receptor Antagonist
MedDRA	Medical Dictionary for Regulatory Activities
MedDRA SOCs	MedDRA System Organ Classes
MeSH	Medical Subject Headings
OAE	Other Adverse Even
OAH	Oral Antihistamine
OSF	Open Science Framework
PRISMA	Preferred Reporting Items for Systemic reviews and Meta-Analyses
RCT	Randomised controlled trials
SAE	Serious Adverse Event
TEAE	Treatment-emerged Adverse Event
TRAE	Treatment-related Adverse Event
URTIs	Upper Respiratory Infections
UTIs	Urinary Tract Infections
WHO	World Health Organization
