# Supplementary material for: Safety Profile of Medications for Allergic Rhinitis: A Meta-Epidemiological Analysis of Completed RCTs from ClinicalTrials.gov
Source: Pharmaceutics. 2026 May 8;18(5):581. doi: 10.3390/pharmaceutics18050581 (PMC13210462; doi:10.3390/pharmaceutics18050581)
Supplement: Supplementary file 1 [file pharmaceutics-18-00581-s001.zip › Supplementary Tables.pdf]

Supplementary Tables

Supplementary Table S1. OAEs significantly more frequent in the high-dose INAH group compared to placebo.

Organ system	OAE	*p value*	RR (95% CI)	OR (95% CI)
**Higher dose of studied INCS vs. Placebo**				
Infections and infestations	UTI	*<0.001*	10.59 (1.35-82.98)	10.89 (1.38-86.17)
**Higher dose of studied INAH vs. Placebo**				
Nervous system disorders	Dysgeusia	*<0.001*	3.58 (2.14-6.00)	4.08 (2.36-7.07)

Abbreviations: OAE – Other adverse event, RR – Relative risk, CI – Confidence interval, OR – Odds ratio, INCS-Intranasal corticosteroids, UTI-Urinary tract infection, INAH – Intranasal antihistamine.

Supplementary Table S2. OAEs significantly more frequent in the high-dose SLIT group compared to placebo.

Organ system	OAE	*p value*	RR (95% CI)	OR (95% CI)
**Higher dose of studied SLIT vs. Placebo**				
Ear disorders	Ear pruritus	*<0.001*	5.75 (3.34-9.89)	6.70 (3.80-11.84)
Gastrointestinal disorders	Oral oedema	*<0.001*	8.59 (2.70-27.34)	11.50 (3.38-39.18)
	Oral pruritus	*<0.001*	5.77 (4.12-8.08)	7.63 (5.30-10.98)
Respiratory, thoracic and mediastinal disorders	Pharyngeal irritation	*<0.001*	3.10 (1.73-5.55)	3.84 (1.99-7.42)
	Pharyngeal oedema	*0.001*	23.14 (1.37-389.48)	24.74 (1.45-423.33)

Abbreviations: OAE – Other adverse event, SLIT – Sublingual immunotherapy, RR – Relative risk, OR – Odds ratio.

Supplementary Table S3. Meta-regression results for factors associated with adverse events across treatment subgroups.

Drug subgroup	Age RR (95% CI)	*p value*	Female RR (95% CI)	*p value*	Duration RR (95% CI)	*p value*
INCS	1.00 (1.00–1.01)	*0.373*	0.81 (0.35–1.86)	*0.622*	1.00 (1.00–1.00)	*0.308*
INCS (high dose)	1.00 (0.98–1.02)	*0.937*	0.89 (0.14–5.50)	*0.899*	1.00 (1.00–1.00)	*0.913*
INAH	1.02 (1.00–1.05)	*0.077*	1.47 (0.06–35.84)	*0.813*	1.00 (1.00–1.00)	*0.101*
INAH (high dose)	1.03 (0.98–1.07)	*0.210*	2.08 (0.03–157.67)	*0.741*	0.98 (0.90–1.07)	*0.611*
INCS + INAH	0.99 (0.95–1.04)	*0.812*	6.66 (0.06–711.34)	*0.426*	1.00 (1.00–1.00)	*0.002*
SLIT	1.01 (1.00–1.02)	*0.071*	0.49 (0.17–1.46)	*0.203*	1.00 (1.00–1.00)	*0.997*
SLIT (high dose)	1.03 (1.02–1.05)	*<0.001*	0.39 (0.17–0.89)	*0.025*	1.00 (1.00–1.00)	*0.034*
OAH	1.02 (0.98–1.06)	*0.300*	0.12 (0.00–27.00)	*0.438*	1.00 (1.00–1.01)	*0.620*
LTRA	0.98 (0.94–1.02)	*0.350*	12.39 (0.31–489.21)	*0.180*	1.00 (1.00–1.01)	*0.142*

Abbreviations: RR-relative risk, CI-confidence interval. Meta-regression analyses were performed using mean age, percentage of female participants, and treatment duration as covariates.
